# Brain and cognitive ageing: The present, and some predictions (…about the future)

**DOI:** 10.1016/j.nbas.2022.100032

**Published:** 2022-02-26

**Authors:** Simon R. Cox, Ian J. Deary

**Affiliations:** a Lothian Birth Cohorts, Department of Psychology, The University of Edinburgh, UK; b Scottish Imaging Network, A Platform for Scientific Excellence (SINAPSE) Collaboration, Edinburgh, UK

**Keywords:** Brain ageing, Structural MRI, Diffusion MRI, Review, Longitudinal studies, Epidemiology

## Abstract

Experiencing decline in one’s cognitive abilities is among the most feared aspects of growing old [53]. Age-related cognitive decline carries a huge personal, societal, and financial cost both in pathological ageing (such as dementias) and also within the non-clinical majority of the population. A projected 152 million people worldwide will suffer from dementia by 2050 [3]. The early stages of cognitive decline are much more prevalent than dementia, and can still impose serious limitations of performance on everyday activities, independence, and quality of life in older age [5], [60], [80]. Cognitive decline also predicts poorer health, adherence to medical regimens, and financial decision-making, and can herald dementia, illness, and death [6], [40]. Of course, when seeking to understand why some people experience more severe cognitive ageing than others, researchers have turned to the organ of thinking for clues about the nature, possible mechanisms, and determinants that might underpin more and less successful cognitive agers. However, that organ is relatively inaccessible, a limitation partly alleviated by advances in neuroimaging. Here we discuss lessons for cognitive and brain ageing that have come from neuroimaging research (especially structural brain imaging), what neuroimaging still has left to teach us, and our views on possible ways forward in this multidisciplinary field.

## Introduction

Looking to the horizon, here’s what we should like to know. What happens to the typical brain as it ages? What are the mechanisms of that ageing? What are the individual differences in brain ageing, and which mechanisms cause those? And we could repeat those questions, mutatis mutandis, by replacing brain with cognitive function, and adding that brain ageing will be a contributor to cognitive ageing (and maybe a bit of the reverse direction, too). These are our concerns in this piece. We recognise the importance, too, of illnesses (from neural and other systems) on the brain and its ageing, and we don’t rule out that some of what we think of as typical ageing might be due to subclinical disorders, and that it might be hard to separate the ageing of the normal brain from the increasing illness burden on the older brain.

## What do we know? (about brain ageing and relations with cognitive capability)

This article is about future-gazing with respect to progress in finding how brain ageing parameters are associated with age-related cognitive changes, especially declines. This risky and error-prone exercise is, we think, best conducted with a short survey of the relatively solid grounding that the subject already has. We also note that far more detailed and lengthy overviews on this specific topic have been written, to which we direct the interested reader (e.g. [35], [65], [49]).

### Structural hallmarks of brain ageing – The mode

The advent of affordable and practical structural MRI studies in particular has led to an impressive body of evidence that provides the foundation of our understanding about the norms of brain ageing. Much of this evidence comes from cross-sectional studies which indicate that both areas in which our brain cell bodies are located (grey matter) and the areas which constitute many connections between brain cells (white matter) exhibit age-related changes. Increasing age across adulthood carries greater risk of global brain atrophy indicated by volume loss of grey and white matter, increase in white matter hyperintensities (WMH; and other markers of small vessel disease; [85], [49]), reductions in the surface area and thickness of the cortex, lower subcortical volumes, and an increase in the size of the ventricles and other intracranial areas which have been vacated by the brain as it shrinks [30], [27], [63], [65], [83], [87].

The brain’s white matter also exhibits other markers of putative ageing-related degradation as indicated by diffusion MRI (dMRI): water molecular diffusion is increasingly unconstrained in older brains, indicative of differences in multiple aspects of neurobiological microstructural environment including – but not limited to – axonal myelination [42]. This is exhibited both its directional coherence (known as fractional anisotropy; FA, which generally goes down with age) and the overall magnitude in all directions (mean diffusivity; MD, which generally goes up with age; e.g. [83], [15], [19], [72], [94]. In addition, it appears that there are regional differences in the susceptibility of the brain to increasing age. This is shown for white matter dMRI parameters, as in those references just cited – which indicate areas most strongly associated with advancing age tend to be cortico-cortical and thalamo-cortical connections rather than projection fibres (such as the corticospinal tract or acoustic radiation), though it is worth noting that there is some evidence of highly restricted white matter regions showing higher FA in older age [54], [91]. These regional differences in ageing are also apparent in other aspects of brain structure. In older adults, greater negative age associations are found in cortical areas such as prefrontal and temporal cortices [30], [93], [12], [35], [65], [28]. In spite of the emphasis traditionally placed upon the hippocampal formation experiencing most ageing [4], [57], it remains moot whether it shows larger linear and non-linear (accelerating) ageing effects than other subcortical structures of the brain such as the thalamus [15], [24], [27], [84]. WMH appear to aggregate most consistently superior to the lateral ventricles, meaning that they are likely to differentially disrupt the normal functioning of specific white matter pathways [86], [88], [18], [95]. Moreover, such effects extend in a penumbra which, while not detectable via T2* / FLAIR acquisitions is apparent on dMRI [89], [46], [51], [56]. Finally, it should be noted that current evidence indicates there are only modest differences (small effect sizes) in the degree to which men and women experience these global and regional aspects of brain structural ageing, particularly for volumetric measures after the reliable differences in head size [26], [68] are accounted for [12], [15], [28], [39], [62].

It is important to note a key feature that differentiates some measures of brain structure from others. Some are frank markers of ageing; i.e., they do not appear, or do not show substantial variability in younger adulthood. Then there are others, whose variability in older age is much more likely to conflate (when cross-sectionally measured) both long-standing, trait-like non-degenerative variation as well as age-related degenerative variance. One examples of the former is WMHs , which almost never appear in the early decades of adulthood. Another is global atrophy (measured by taking total brain volume as a function of intracranial volume). This second measure reflects how much brain one currently has relative to how much one had when – at maximal healthy size – the brain filled the intracranial vault [81]. Examples of structural imaging measures that conflate ageing and non-neurodegenerative variance in cross-sectional evaluation include cortical characteristics, unadjusted tissue volumes, and diffusion metrics. There are clear individual differences across the life course in these latter measures, making it hard to infer whether and to what degree neurodegenerative processes are influencing the differences between people using cross-sectional data alone. That is, if one obtains, say, measure of white matter tract FA from a person that suggests a low value, one does not know whether they were always like that, or whether their brain has deteriorated with age. One clear way around this issue is to use longitudinal data. The use of cross-sectional information to make inferences about brain and cognitive ageing has been strongly criticised in some quarters: it is unable to adequately approximate the dimensionality and time-dependent dynamics of ageing [29], [64], [70], [71]. Yet, using multiple measures of the same people over time, one can more explicitly model both the individual differences in level as well as distinctly model a person’s ageing-related trajectory over time. We agree that longitudinal data are critical for providing more direct insights into ageing-related processes; nevertheless, no method is flawless. Without due care, pseudo-longitudinal designs are potentially susceptible to the same cohort effects that affect cross-sectional studies of ageing. Moreover, non-random attrition is an unavoidable consequence of seeking to bring in the same older people on multiple occasions (where estimates are biased against the least healthy, who are often most likely not to return). As such – particularly given the scarcity of valuable longitudinal data on brain and cognitive ageing [58] - we are of the view that cross-sectional data should not be overly eschewed for its limitations, but that these studies, too, can offer valuable insights into ageing processes alongside longitudinal methods. In particular, it is currently impossible to build a complete picture of brain development and ageing from purely longitudinal data from the same individuals. Thus, we predict the continued growth of innovative projects to incorporate cross-sectional, pseudo-longitudinal and longitudinal designs across cohorts to improve our picture of modal brain ageing.

### Structural hallmarks of brain ageing – Individual differences

**Fig. 1:**
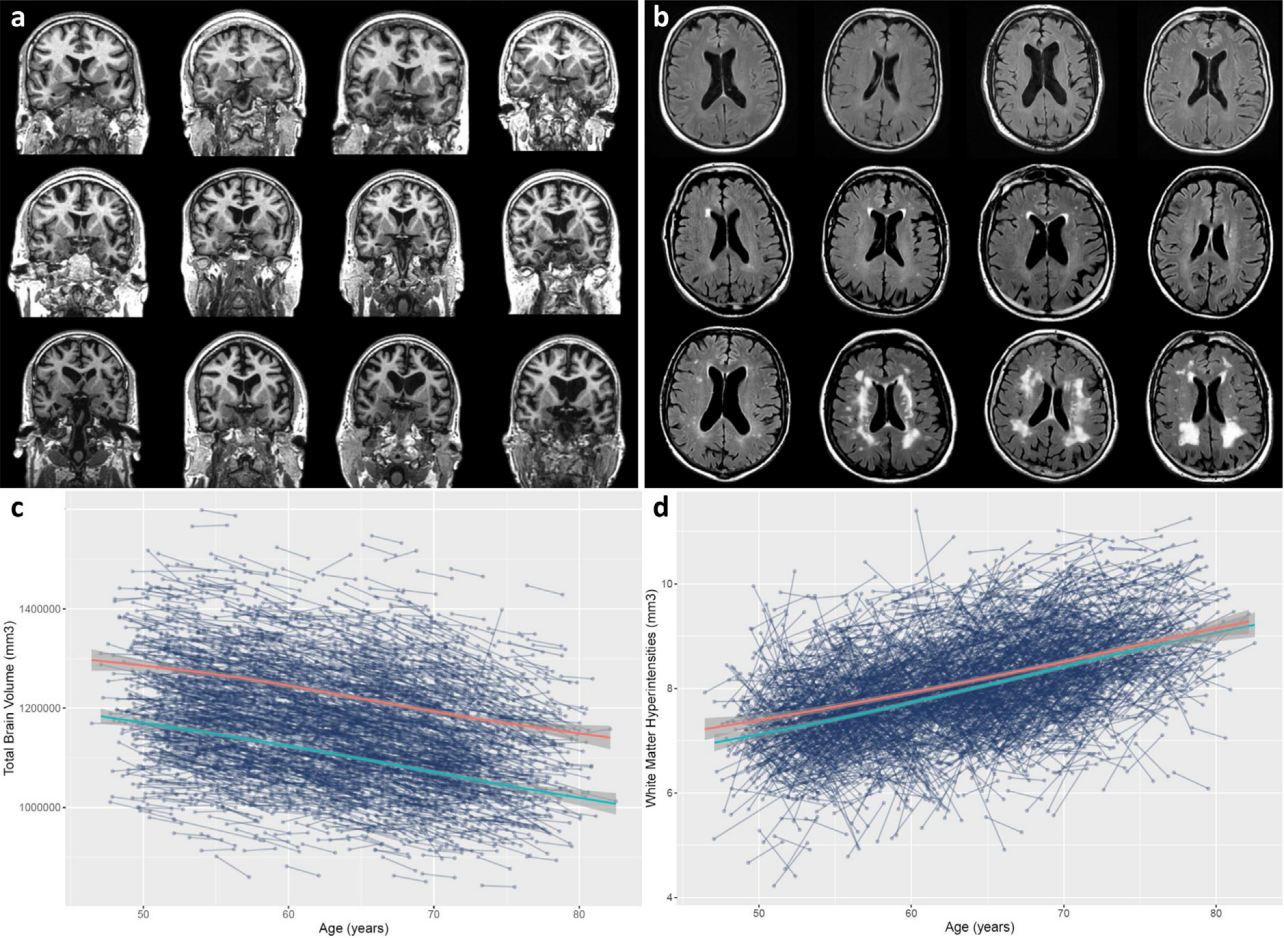
Individual differences in brain structural ageing hallmarks. **a)** Different participants, ordered from top left to bottom right by severity of global atrophy (parenchymal volume fraction: total brain volume / intracranial volume). MRI-visible markers include global cerebral atrophy, cortical thinning, and ventricular enlargement alongside sulcal widening as CSF (black) replaces brain tissue. **b)** T2 FLAIR sequence shows increasing severity of white matter hyperinensities (volume increasing from top left to bottom right). Participants from this figure come from the Lothian Birth Cohort 1936 wave 2: community-dwelling adults aged ∼ 73 years at time of scanning. Participants between panels do not correspond. **c)** Longitudinal total brain volume measurements from 3189 individuals in UK Biobank (Field ID: 20510) with local polynomial regression lines (loess) for females (red) and males (blue). **d)** Longitudinal white matter hyperintensity volume measurements from 3152 individuals in UKB Biobank (Field ID: 25781) with local polynomial regression lines (loess) for females (red) and males (blue). (For interpretation of the references to colour in this figure legend, the reader is referred to the web version of this article.)

Whereas it is important to be able to characterise modal brain ageing, our ability to understand how and why people experience ageing differently relies upon the characterisation of individual differences. Is brain ageing, as described above, inevitable for everyone? Moreover, accounting for some of the variability in cognitive ageing with brain measures necessitates an understanding of the variability in these brain hallmarks too. Fig. 1 is helpful in this regard: first, it might be useful as a visual key to the progression of some of the different brain hallmarks discussed above (ordered least-most severe global atrophy, and WMH volume, respectively, from top left to bottom right). One can see, for example, as one looks at each brain in turn, progressively larger ventricles, thinner cortex, and lower volumes of both grey and white matter alongside increasing WMH. Second, and most importantly, it also shows in stark terms the variability with which older individuals experience ageing: all participants in the figure were scanned at around age 73 (all being born in 1936), most were healthy, and none of them had been diagnosed with a neurodegenerative condition, including dementia. Thus, it is a compelling reminder that brain ageing is not a foregone conclusion, and why one must also delve into the individual differences of brain ageing to understand both the cognitive sequelae of brain ageing, and the factors which might identify those at greatest risk, or identify targets for future treatments or interventions. The latter is too large an area to summarise here, though we now turn briefly to the former, before addressing ways in which new methods and approaches might be best applied to understanding the individual differences which we expect might be most informative in ageing research discovery.

### Brain and cognitive ageing

When researching the brain, our own personal interest arises because of its potential as a biologically tractable marker of the things it does. That is, we are looking at it as the organ of thinking because we think different facets will give otherwise unavailable information about the organ’s functioning. We acknowledge, too, that there are many other things that the brain does (somatosensory, motor, autonomic regulation etc.), but these are less directly within our sphere of interest than ageing of complex cognitive functioning. We have written about what we currently know about how the brain’s structure fits with cognitive ageing in detail elsewhere [23], and our take-home messages here are four-fold. First, many of the global structural hallmarks of brain ageing appear to carry some unique information about cognitive differences. That is, for example, that in a multivariate sense, each of global atrophy, cortical characteristics, white matter volumes, WMH and white matter dMRI measures tends to predict a small but unique amount of variance in cognitive differences, explaining up to about 20% in older samples [13], [67]. Given these are fairly gross measures of only one aspect (structure) of the brain, and that no correction has been made for measurement error, this might be considered quite a large amount. Further, we found that dMRI metrics only explained unique variance in general cognitive functioning among older participants (these were extremely small and non-significant in those in middle age; [13]. We take this to indicate that these measures probably contain a greater proportion of neurodegeneration-related variability as age increases (as opposed to normal variation – see point above). Second, we and others find that those parts of the brain apparently most susceptible to ageing are also those that are the best candidates for underpinning our most complex cognitive functions. For example, patterns of grey and white matter regional correlates of general intelligence show marked similarities with cross-sectional age associations [15], [13], [50], [93]. Third, we and others have using longitudinal data to test more directly the idea that specific regional brain changes are related to cognitive declines. Overall, longitudinal declines in cognitive functioning are associated with declines in global brain structural measures at the whole-brain and tissue-specific levels, implicating both grey and white matter structure [58]. Similarly, in longitudinal analyses in the Lothian Birth Cohort 1936, we found that a factor of shared cortical volumetric aging – more strongly represented by frontal and temporal regions – was correlated with cognitive ageing across multiple domains [13]. Finally, we want to be clear that cognitive functions are diverse, and that the regionally heterogeneous cytoarchitectural and hodological makeup of the brain necessitates a degree of functional diversity. Nevertheless, given the importance of modelling within-person change over time, and that it has been robustly shown that declines across higher-order cognitive domains become increasingly statistically indistinguishable with increasing age [79], focussing on general cognitive ageing is highly valuable, and also allows one to accurately assess what is not general (uniquely domain-specific). In other words, the statistical modelling of domain-specific cognitive decline in older age is nearly analogous to modelling general cognitive declines. To paraphrase Rabbitt [61], it *does* all [or a substantial proportion of it] go together when it goes. It remains to be seen whether the patterns of longitudinal brain changes themselves also de-differentiate statistically (as we and others have shown cross-sectionally; [15], [21] to mirror this de-differentiation in cognitive ageing, and whether the modest domain-specific variance in cognitive ageing can be accounted for by specific facets of brain change, and by unique environmental and genetic predictors.

## Where do we go? (regarding brain ageing and its relations with cognitive capability)

We are aware of the dangers of answering this question; this was expressed, as follows, by the Danish politician Karl Kristian Steincke [76] in a Danish parliamentary howler he noted in his autobiography: “Det er vanskeligt at spaa, især naar det gælder Fremtiden”. But we can try our best. As a start, we can divide the future into categories, which we shall exemplify below. Thus, there are, first, some methods emerging whose results will be harvested in the next several years. Second—and we can say less about this, obviously—there will be new methods which we can’t yet foresee. For example, imagine our having to speculate about the future of understanding the structure of DNA prior to X-ray crystallography. As priest and author of the Art of Thinking, Ernest Dimnet wrote, “Too often we forget that genius, too, depends upon the data within its reach, that even Archimedes could not have devised Edison's inventions.” Third, we can make suggestions for better practice, whether that be with currently-available or future methods.

### Emerging methods

Here, we reflect on the recent past, and how we are using methods to address brain and cognitive imaging differences in the Lothian Birth Cohorts that were not available—or feasible—when they started in the late 1990s. For example, we have adopted the following [78]: GWAS, polygenic scores, molecular genetic correlations, gene sequencing, genome-wide epigenetic testing, gene expression, lipidomics, metabolomics, telomere length testing and epigenetic clocks, analysis of various biomarkers (e.g. inflammatory), retinal vessel topographic analysis, carotid artery Doppler ultrasound, a detailed microscopic and biochemical analysis of post-mortem tissue, stem cell development and analysis, identification of environmental exposure history using geographical information systems. Some of these, of course, are done as part of consortia, using meta-analytic and mega-analytic statistical analyses. Our statistical analytic methods have kept up, using, for example growth curve modelling in a structural equation modelling framework, linear mixed models, and various forms of machine learning. And we have learned and adopted correction methods for type 1 errors as they have developed, but we suspect and hope that we will be largely freed of those concerns - even for small effects - as sample sizes grow, leading – we expect – to an emphasis on effect sizes and out-of-sample replicability thereof (see sections below). Of course, other biological methods and markers will emerge—some of which will capture variance in brain and cognitive ageing, and some not—and we can’t list or even foresee them all here. However, we now mention some in the realm of brain imaging.

One such example arises from the more complex parameterisations of multi-shell diffusion data (such as Neurite Orientation Dispersion and Density Imaging; NODDI; [92] can potentially increase the specificity of our white matter imaging biomarkers to fewer facets of the microstructural environment (note, some authors caution that some of the modelling assumptions may lead to some forms of bias; [41]). Though we found that the overall age sensitivity was not substantially stronger than in conventional dMRI white matter measures in the cross-sectional UK Biobank [15], more recent longitudinal analyses indicate that NODDI parameters carry unique information about brain development and ageing beyond more conventional indices [94]; [10]. We further observe that the evaluation of an emerging method should also be assessed based on biological tractability (as well as incremental external / criterion validity).

One arena in which NODDI indices may contribute further is via integration into the brain structural connectome from structural and diffusion MRI. This offers the potential to understand brain ageing across scales by estimating each individual’s connective white matter pathways between distal cortical and subcortical sites (in native space) and weighting each set of site-to-site connections according to a measure of choice (e.g. number of tractographic streamlines, average FA, MD or NODDI parameter across all streamlines between each region, or ‘node’, pair). It therefore provides measures of a great many more connections in native space than either conventional tractography methods (many fewer, major pathways) or Tract-Based Spatial Statistics (which focusses only on the very small proportion of skeletonised white matter that overlaps well across all subjects in standard space). The connectome allows focus on (1) the diffusion properties of connections related to specific regions or networks of interest, and/or (2) global or network-centric measures of network topology, by computing graph-theoretical descriptors (e.g. [8]). Both are attractive given the rise in network-centric concepts of brain structure and function, and the former approach (many site-to-site connections) offers a potential solution to the possibility that judgements about regional differences in age sensitivity within white matter are biased by the relatively restricted amount of white matter considered when measuring larger but fewer pathways, as in more conventional methods. For example, using a fairly restricted 27 white matter pathways in UK Biobank, we found that thalamic radiations had among the strongest age associations across diffusion measures [15]. However, this limited number of pathways did not include many other subcortical pathways; connectome-derived white matter diffusion measures indicated that caudate connectivity, too, was among the strongest age associations [7]. Notably, as with other MRI-based approaches, particularly at lower field strengths and lower resolutions, the higher fidelity with which one may wish to map nodes (e.g. cortical and subcortical regions) can lead to an increase in noise. This is particularly true when higher resolution atlases are used [22], [25] – and is complicated by the fact that there remains no definitive / agreed-upon way to parcellate the brain for optimal representation of cytoarchitecture / function / hodology. Finally, with respect to global graph theoretical measures that can be used to summarise global or sub-network topological properties from the connectome (of which there are many), it appears that there are situations in which the graph theoretical measures of a brain network are highly collinear with each other, and with the network weighting from which they are derived [2], [7], [66]. It will be valuable here, too, to establish whether these measures carry unique information about brain ageing, beyond that which can already be described by existing measures.

There will of course continue to be a strong tradition in developing new MRI acquisitions and new methods for processing what has been acquired. Though not especially new, we and others (e.g. [54]) have noted that susceptibility-weighted MR imaging (swMRI) holds the potential to better understand vascular contributions to brain structural ageing. It is now well-established that there are modest but robust associations between higher vascular risk (e.g. smoking, hypertension, hypercholesterolemia, diabetes) and both cerebrovascular complications [20] and poorer brain structural parameters in relatively healthy community-dwelling adults [14], [55], [75], [82]. The ability to quantify aspects of cerebrovascular disease in swMRI – thanks to the enhanced conspicuity of medium and large cerebral vessels – holds promise for producing venogram segmentations and enhancing microbleed segmentations from other sequences, which in turn will further refine our understanding of the specific vascular contributions (beyond macrostructural properties such as volumetry) that vascular risk factors make to brain structural ageing (see [48]).

The use of machine / statistical learning to identify brain features that can optimise prediction of chronological age has garnered substantial attention in recent years [16], [44]. That these paradigms require stability in out-of-sample performance is a huge strength in post-replication-crisis academia, and one that will continue to permeate other aspects of ageing-related fields [90]. Though the approach is not without appeal as a valuable and intuitive biomarker [16], it is important to bear in mind the several potential limitations too. For example, there are statistical and inferential challenges levelled at Brain Age prediction [9], [74], and there remains a tension between losing regional and tissue-specific fidelity versus the potential reductive utility of brain age acceleration as a diagnostic tool. The inability to peek inside the black box is a criticism that can also be levelled at deep learning approaches, whereby it has been previously hard to judge the differential importance of different aspects of, say, a standard T1-weighted volume for predicting age. The implementation of saliency mapping methods (such as variants of backpropogation and gradient class activation mapping; Grad-CAM) have been adapted to work on 3D MRI data, offering greater insight into the most salient features for learning and classifying brain age differences [73]. Nevertheless, interpreting the retained (or retrieved) information about the unique contributions of different brain regions to brain age prediction cannot be undertaken in the conventional neuroscientific tradition. Valences of some regions can be negative, for example, when few brain volumes grow into older age. This, of course, is because that is not the job we are asking statistical learning to accomplish – but nevertheless, it recapitulates the tension between understanding which areas might be biologically important (e.g. for supporting specific processes or whose changes are potentially related to declines in those processes) and prediction / classification. The latter is where we judge this particular class of approaches will offer more valuable contributions, though any future clinical implementation of such methods for predicting important outcomes (such as steeper future brain ageing or likelihood of progression to dementia) must exhibit excellent prediction accuracy (sensitivity and specificity), and offer quantifiable gains over more conventional methods and markers.

### Bridging the explanatory gap and adopting new methods that arise

Cognitive ability is one of the read-outs of the brain’s functioning. Brain imaging indices—structural and functional—might mediate between brain biology and cognitive functioning. There is a problem in closing the explanatory gap between brain and cognition and even laying out what age does to the brain, i.e. that getting at human brains in vivo is almost impossible. Therefore, it is difficult to explore causes of brain and cognitive differences at the cellular level. This has much to do with issues with specificity of MRI estimates of brain structure and function. This includes, for example, the limitations of dMRI’s specificity to multiple facets of cellular ageing in white matter (which remains more than just axonal myelination), the precise cellular makeup of a ‘bigger brain’ that might explain the modest but robust association with general cognitive ability, and what specifically is lost or changes during the ageing process as related to the macro- and microstructural estimates derived from MRI. This makes precise explanations of the cellular and molecular underpinnings of ageing-related phenomena challenging. Even post mortem data necessitates an indirect extrapolation, and there remains a clear difficulty in reliably ascribing macro-structural in vivo observations on ageing - especially longitudinal ones - to differing levels of information on the many molecular and cellular hallmarks of brain ageing (e.g. [52]). Still, we note cause for optimism here. Whereas it is currently impossible to have longitudinal histological data, there has been a substantial increase in the availability of neurobiological resources that can allow researchers to bridge multiple levels of brain data. Albeit still indirect, the availability of data on gene expression (e.g. [31]), neurotransmitter systems [36] histology [59], among others – critically in a format that allows direct mapping to the same standard space as macrostructural MRI data – will offer a huge boost to the inferential arsenal via which we can understand the underlying biological and molecular bases of brain ageing.

## General principles for doing better with what we have now

### Expect small effects

There is a realisation that complex traits—such as individual differences in cognitive ageing—are influenced by many factors—environmental and genetic [17]. Typically, these factors will each have small effects [32], [33]. This mandates our having well-powered studies that can cope with multiple small effects, with multiple covariates, and whose data can be analysed with methods that have some capability to determine causal directions; e.g. directed acyclic graphs with structural equation modelling [34] and Mendelian randomization. Studies in the area need unfamiliarly large numbers and longitudinal data, e.g. such as those from the Human Connectome Project (www.humanconnectome.org), the Rhineland Study (https://www.rheinland-studie.de/), UK Biobank (www.ukbiobank.ac.uk), and even larger. This will prevent the large numbers of non-replications such as those in functional brain imaging and candidate gene studies. Large sample sizes will also present new challenges in terms of deconfounding MRI data; small nuisance variables can now more reliably account for small portions of variance not linked to the effect of interest (e.g. [1]) though it is important to note that in some scenarios, large-scale deconfounding does not perform much better than a fewer ‘simple’ confounds, and this recent work has highlighted how much care will need to be taken in confound selection depending upon the modality and the types of outcomes under investigation.

### Recruit appropriately and diversely

Though there have been calls to focus on mid-life to understand cognitive and brain ageing (under the assumption that this is when early portents of later decline may be detectable; [69], it is important not to overlook the substantial underrepresentation of the oldest old in large imaging studies. This is partly a practical issue (and the frailest and least healthy will be less likely to tolerate lying still for long), but the lack of large sample multi-modal imaging data (especially longitudinal) on participants aged 70+ (and even more so for those aged 80 + ) will also be highly beneficial as these are the participants at greatest risk of dementia and cognitive decline (and a time at which potential stratification of ‘normal’ and pathological brain ageing may be most clearly detectable due to the large changes possible over a shorter period (enabling more reliable detection of change). It will also be important to ensure that future samples allow greater power. That many research samples – and many of the neuroimaging samples described here, including our own Lothian Birth Cohort – are WEIRD (from Western, Educated, Industrialised, Rich and Democratic societies), is well known [37]. When taken alongside the fact that participation in research is also selective [47], [38] and that analysis of ‘completers only’ will further bias longitudinal estimates of change away from trajectories of the less healthy or able (e.g. [12]), the evidence base for our understanding of brain ageing is doubly restricted. We thus agree with those who advocate for both more diverse and inclusive recruitment [11], [45], [83], and to employ statistical methods to characterise and account for the biases present in initial recruitment self-selection and via attrition and missingness, longitudinally.

### Openness in data collection

The brain thinks (and feels, and wills, and co-ordinates etc.), and it thinks more or less well, and it generally thinks less well with age. At present, the variance accounted for in thinking by brain imaging variables is at best about a fifth of the whole. That suggests there are more and better ways in which brain functioning can be characterised and in which we can account for ageing effects. What to do, apart from using current methods? With a large and diverse sample, and one that captures variance in the outcomes of interest, we can at least advise comprehensiveness in the collection of variables that are likely contributors. We also strongly advocate the adoption of good practices such as out-of-sample replication in ageing neuroscience, which is partly replacing the desire simply to explain as much variance as possible in a given dataset (thus avoiding overfitting, effect size inflation and other such pitfalls; [90]; this will become increasingly possible as sample sizes and datasets continue to grow and become accessible to the wider scientific community.

In addition, there should be a looking-out for growing points in substantive and methodology areas. Some of these will be in one’s own area of specialisation and some not, so one has to be collaborative. The collaboration has to work; there is no point in collaborating with a specialism with which one cannot shake hands; there should be no understanding gap in the collaboration. Not all new variables will make a contribution; there will be some duds and some winners, and we hope that growing support for the publication of null findings will support the critical need to help the wider scientific community sort one from the other. One also need to keep in mind what one is trying to do: there is a place for characterising both modal and individual differences in brain ageing. That is, only some of the important factors in making brains work and in which brains age will have parameters that are relevant for individual differences in cognitive performance and cognitive ageing.

Part of the openness is the obvious fact—and its consequences once appreciated—that the brain is made of, and supported by, stuff that the body is made of. Therefore, don’t ignore leads/relevance from general ageing science [16]; it will provide variables that can be assessed and related to brain ageing. Therefore, mind-stretching multi-disciplinarity is essential, both with respect to substantive fields and methods. Part of this will be, for example, vascular factors, because some of brain ageing is vascular ageing. The contributions of glia and the in-head immune system should be assessed too.

## Conclusion

Our recommendations are that, if we are to understand what happens to brains and thinking as people age, then we must expect multiple small effects, we must recruit much larger and more diverse samples, and we must look—and be on the lookout—much more widely for explanatory variables. We note the current position of GWAS studies of complex traits—including brain variables and thinking skills: there are many, many significant genetic variants contributing to these traits and the effect sizes are very small indeed. This could stultify efforts to find mechanisms [43], [77]. This could make it seem like progress is nearly impossible because to follow up and integrate so many small effects’ mechanisms would be intractable. But it wasn’t much more than a decade ago that GWAS would have sounded like science fiction. Therefore, we should not be put off by clear advances that also seem to land us in a thicket. As the journalist Sidney J. Harris wrote, “A cynic is not merely one who reads bitter lessons from the past, he is one who is prematurely disappointed in the future.”

Brains age – those brain areas most strongly linked with ageing also appear to be those that are most strongly linked to our most complex cognitive abilities and perhaps also to potential determinants such as vascular risk. There are lots of new avenues, there is huge potential, and there is a need to link with other levels of explanation which we are beginning to see results from. Within our recommended multi-disciplinary openness, there should be a good tie with brain ‘outputs’, such as cognition. The question of how the brain ages and why people differ in that sadness interests us because we see some thinking skills decline from early and mid-adulthood, and we see some declining more than others. Finding the paths from explanans to explanandum means being comprehensive about what might comprise both, and measuring them robustly.

## Declaration of Competing Interest

The authors declare that they have no known competing financial interests or personal relationships that could have appeared to influence the work reported in this paper.
